# A Retrospective Study of Best Supportive Care as Initial Treatment for Oral Squamous Cell Carcinoma

**DOI:** 10.7759/cureus.89319

**Published:** 2025-08-04

**Authors:** Yuko Saito, Hideaki Hirai, Ryota Kobayashi, Aki Kasahara, Tetsuo Kiguchi, Yoshimasa Sumita, Atsushi Uenoyama, Kei Tomihara

**Affiliations:** 1 Division of Oral and Maxillofacial Surgery, Faculty of Dentistry and Graduate School of Medical and Dental Sciences, Niigata University, Niigata, JPN

**Keywords:** best supportive care, initial treatment, older people, oral squamous cell carcinoma, primary case

## Abstract

Objective

Patients with oral squamous cell carcinoma (OSCC) may choose best supportive care (BSC) as initial treatment over active treatment for several reasons. However, no previous reports have compared the clinical characteristics of OSCC patients opting for BSC as initial treatment with those receiving standard treatment. This study retrospectively analyzed these differences to identify the factors influencing this decision.

Materials and methods

This retrospective study was conducted at Niigata University Medical and Dental Hospital, using medical records within January 2018-December 2022 (follow-up until June 2023). Among 135 patients with primary OSCC, received BSC (BSC group) and underwent standard treatment according to the disease stage (non-BSC group). Data included demographic and tumor characteristics, Eastern Cooperative Oncology Group Performance Status (ECOG-PS), underlying diseases, neutrophil-lymphocyte ratio (NLR), albumin levels, living situation (with family, in a care facility, or alone), rationale for BSC selection (untreatable due to poor general condition or no desire for treatment), overall survival (OS) rate, and disease-specific survival (DSS) rate. Age, NLR, and albumin levels were assessed using Student’s t-test. Stage classification, ECOG-PS, and living situation were analyzed using the chi-square test. OS and DDS were calculated using the Kaplan-Meier method and compared using the log-rank test. Multiple logistic regression analysis identified factors associated with BSC.

Results

The BSC group included 11 males and 13 females (mean age: 84 years; range: 47-92). The non-BSC group included 67 males and 44 females (mean age: 67.4 years; range: 31-89). Female predominance was higher in the BSC group (male-female ratio: 1:1.2) than in the non-BSC group (1:0.7). Primary sites were the lower gingiva in the BSC and the tongue in the non-BSC group. The BSC group exhibited higher NLR (p=0.50) and a significantly lower albumin level (p<0.001). Stage classification (I/II vs. III/IV) and ECOG-PS (0-1 vs. 2-4) differed significantly between groups (p<0.001). Cardiovascular disease was the most frequent comorbidity in both groups. Living situation exhibited no significant differences (p=0.99). The cumulative one-year OS rate in the BSC group was 29.8%, compared to 97.2% and 81.5% in the non-BSC group at one and five years, respectively. The cumulative one-year DSS rate in the BSC group was 31.9%, and 97.2% and 82.8% at one and five years in the non-BSC group were p<0.001 for OS and DSS. Age (OR: 1.20, 95% CI: 1.10-1.36; p=0.0008), stage classification (I/II vs. III/IV) (OR: 13.79, 95% CI: 2.34-137.75; p=0.0026), psychiatric disorder (OR: 8.73, 95% CI: 2.34-137.75; p=0.021), albumin level (OR: 0.03, 95% CI: 0.002-0.24; p=0.003) were factors in BSC. Among patients aged ≥75 years, stage classification (I/II vs. III/IV) (OR: 19.08, 95% CI: 2.51-321.07; p=0.0027), albumin level (OR: 0.003, 95% CI: 9.0×10^-6^-0.098; p=0.01) remained significant predictors of BSC.

Conclusion

Patients opting for BSC tended to be older. Stage classification and albumin level were key factors in BSC across all patients, including those aged ≥75 years. Early diagnosis and timely intervention are vital to improve treatment opportunities. Collaborative discussions among patients, families, and healthcare providers are crucial to develop individualized care plans, ensuring that patients receiving BSC can approach end-of-life care with dignity.

## Introduction

Best supportive care (BSC) focuses on maintaining and improving quality of life (QOL) by reducing the physical pain caused by cancer and the side effects of treatment in patients who are unsuitable for conventional treatment or do not desire aggressive cancer treatment [1]. Early integration of supportive care into cancer treatment exhibited a positive impact on QOL and perception of disease [2]. Postoperative dysfunction following extensive surgery and complications related to systemic chemoradiotherapy are inevitable in advanced oral squamous cell carcinoma (OSCC). Recurrence or metastasis in such cases may prompt a transition to BSC when conventional therapies fail. Patients with head and neck cancers, including oral cancer, experience substantial physical, psychological, and functional burdens that significantly impact QOL. Symptoms often disrupt essential functions such as eating, speaking, and breathing, and are often accompanied by alterations in facial appearance, taste, hearing, and swallowing [3]. Addressing these through comprehensive supportive care is crucial for maintaining and improving QOL. Despite this, various factors may influence the decision to choose BSC, raising concerns about whether their care needs are adequately met. Investigating the characteristics of BSC patients, the comorbidities, life circumstances, reasons for choosing BSC, and prognosis can provide valuable insights to guide treatment strategies and potentially delay or avoid the need for BSC. However, to the best of our knowledge, no reports have investigated the background and prognosis of patients who choose BSC as an initial treatment strategy. Therefore, this study aimed to investigate the clinical and demographic characteristics of patients with OSCC who received BSC at our department and to identify challenges in preventing early transition to BSC.

This article was previously presented as a poster at the 68th Congress of the Japanese Society of Oral and Maxillofacial Surgeons on November 11, 2023.

## Materials and methods

Study design and setting

This retrospective study was conducted at Niigata University Medical & Dental Hospital. Patient data were reviewed from January 2018 to June 2023 to investigate the clinical characteristics of patients with OSCC and to compare these characteristics between patients with BSC and non-BSC.

This study was approved by the Ethics Committee of Niigata University (approval number: 2023-0092) and was in compliance with the Helsinki Declaration. The requirement for informed consent was waived owing to the retrospective design and use of anonymized data.

Participant selection

Among the 135 patients with OSCC who visited our department between January 2018 and December 2022, those aged ≥20 years at their initial examination were included in the review. Patients were excluded if they had received treatment at other facilities or lacked a histopathological diagnosis of SCC.

Data collection and management

Data were extracted from electronic medical records by trained researchers using a standardized protocol to ensure consistency and reliability. The electronic medical records were de-identified. This study focused on age, sex, primary tumor site, tumor/node/metastasis (TNM) stage (Union for International Cancer Control, 8th edition), stage classification, Eastern Cooperative Oncology Group Performance Status (ECOG-PS), underlying diseases, neutrophil-to-lymphocyte ratio (NLR), and albumin levels at initial examination, living situation (lived with family, in a care facility, or alone), rationale for BSC selection (untreatable due to poor general condition or no desire for treatment), overall survival (OS) rate, disease-specific survival (DSS) rate, and factors associated with BSC.

Statistical analysis

All statistical analyses were performed using the JMP Pro (version 18.2.1; SAS Institute Inc., Cary, NC). Statistical significance was set at p<0.05. Age, NLR, and albumin levels were assessed using Student’s t-test. Stage classification, ECOG-PS, and living situation were analyzed using the chi-square test. Survival rates were calculated from the time of diagnosis until the end of the follow-up period, and Kaplan-Meier curves were plotted. The follow-up period ended in June 2023. Log-rank tests were used to determine statistical differences. Multivariate logistic regression analysis was used to extract factors in BSC.

## Results

Patient characteristics

The BSC group consisted of 24 patients (17.8%), including 11 males and 13 females, aged 47-92 years (mean age: 84 years). The non-BSC group included 111 patients (82.2%), including 67 males and 44 females, aged 31-89 years (mean age: 67.4 years). Patient characteristics for both groups are summarized in Table 1. The mean age in the BSC group was significantly higher than in the non-BSC group (p<0.001). The male-to-female ratio was 1:1.2 in the BSC group and 1:0.7 in the non-BSC group, indicating a female predominance in the BSC group. The most common primary site was the lower gingiva in the BSC group (seven patients, 29.1%) and the tongue in the non-BSC group (58 patients, 52.3%).

**Table 1:** Patient characteristics BSC: best supportive care

Variables	BSC group	Non-BSC group	p-value
	n=24 (%)	n=111 (%)	
Sex			
Male	11 (45.8)	67 (60.4)	
Female	13 (54.2)	44 (39.6)	
Male:Female	1:1.2	1:0.7	
Age (years)	47–92	31–89	
Mean	84	67.4	<0.001
Median	86.5	69	
Primary site			
Tongue	6 (25)	58 (52.3)	
Lower gingiva	7 (29.1)	17 (15.3)	
Buccal mucosa	4 (16.7)	11 (9.9)	
Upper gingiva	3 (12.5)	14 (12.6)	
Hard palate	2 (8.3)	0 (0)	
Floor of mouth	1 (4.2)	7 (6.3)	
Others	1 (4.2)	4 (3.6)	

TNM stage

In the BSC group, tumors were classified into T2 (n=6, 25%), T3 (n=3, 12.5%), T4a (n=13, 54.2%), or T4b (n=2, 8.3%). Lymph nodes were classified into N0 (n=8, 33.3%), N1 (n=5, 20.8%), N2b (n=7, 29.2%), N2c (n=1, 4.2%), or N3b (n=3, 12.5%). One patient (4.2%) was classified as M1 (T4aN2bM1).

In the non-BSC group, tumors were classified into T1 (n=46, 41.4%), T2 (n=41, 37%), T3 (n=9, 8.1%), T4a (n=12, 10.8%), and T4b (n=3, 2.7%). Lymph nodes were classified into N0 (n=90, 81.1%), N1 (n=8, 7.2%), N2b (n=6, 5.4%), N2c (n=1, 0.9%), and N3b (n=6, 5.4%). All patients in the non-BSC were classified as M0.

A summary of the stage classification is tabulated (Table 2). In the BSC group, 83.3% (n=20) had advanced stage III/IV disease, whereas in the non-BSC group, 70.3% (n=78) had early stage I/II disease. Comparative analysis of stage classification (I/II and III/IV) revealed a statistically significant difference between the BSC and non-BSC groups (χ2=23.78, df=1, p<0.001, φ=0.42).

**Table 2:** TNM stage BSC: best supportive care; TNM: tumor/node/metastasis

Stage	BSC group	Non-BSC group	p-value
	n=24 (%)	n=111 (%)	
I	0 (0)	45 (40.6)	<0.001
II	4 (16.7)	33 (29.7)	
III	2 (8.3)	11 (9.9)	
IVA	12 (50)	15 (13.5)	
IVB	5 (20.8)	7 (6.3)	
IVC	1 (4.2)	0 (0)	

ECOG-PS

ECOG-PS scores of 2-4 were observed in 45.8% (n=11) of patients in the BSC group and 2.7% (n=3) in the non-BSC group (Table 3). Comparative analysis of ECOG-PS scores (0-1 and 2-4) differed significantly between the two groups (χ2=39.49, df=1, p<0.001, φ=0.54).

**Table 3:** Eastern Cooperative Oncology Group Performance Status (ECOG-PS) BSC: best supportive care

ECOG-PS	BSC group	Non-BSC group	p-value
	n=24 (%)	n=111 (%)	
0	3 (12.5)	96 (86.5)	<0.001
1	10 (41.7)	12 (10.8)	
2	2 (8.3)	3 (2.7)	
3	6 (25)	0 (0)	
4	3 (12.5)	0 (0)	

Underlying diseases

In the BSC group, cardiovascular disease (n=12, 50%) was the most common, followed by psychiatric disorder (n=11, 45.8%) and endocrine metabolic disease (n=10, 41.7%). In the non-BSC group, cardiovascular disease (n=67, 60.4%) was also the most common, followed by endocrine metabolic disease (n=46, 41.4%) and digestive system disease (n=28, 25.2%) (Table 4). The number of patients with underlying diseases was 24 patients (100%) in the BSC group and 106 patients (95.5%) in the non-BSC group (χ2=1.12, df=1, p=0.29, φ=0.09). These results included duplicate cases.

**Table 4:** Underlying diseases BSC: best supportive care

Underlying diseases	BSC group	Non-BSC group
	n=24 (%)	n=111 (%)
Cardiovascular disease	12 (50.0)	67 (60.4)
Psychiatric disorder	11 (45.8)	9 (8.1)
Endocrine metabolic disease	10 (41.7)	46 (41.4)
Cerebrovascular disease	7 (29.2)	5 (4.5)
Digestive system disease	4 (16.7)	28 (25.2)
Respiratory disease	3 (12.5)	10 (9.0)
Autoimmune disease	0 (0)	6 (5.4)
Others	21 (87.5)	72 (64.9)

NLR and albumin evaluation

**Figure 1:**
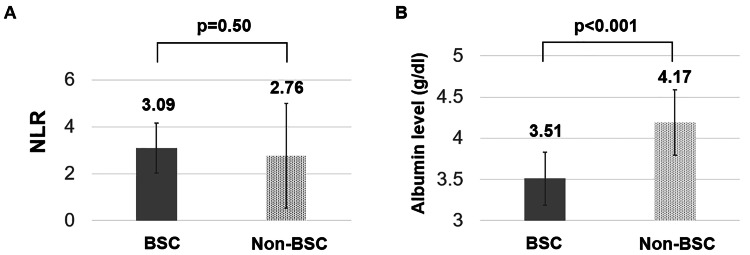
Neutrophil-to-lymphocyte ratio (NLR) and serum albumin levels in the BSC and non-BSC groups at the initial examination (A) The mean±standard deviation of NLR is 3.09±1.06 in the best supportive care (BSC) group and 2.76±2.26 in the non-BSC group. There is no significant difference between the two groups (p=0.50). (B) The mean±standard deviation of serum albumin levels is 3.51±0.32 g/dL in the BSC group and 4.17±0.40 g/dL in the non-BSC group. A significant difference is observed between the two groups (p<0.001).

The mean±standard deviation of NLR (Figure 1A) and serum albumin levels (Figure 1B) at the initial examination were evaluated. While no significant difference was observed in NLR between the two groups, serum albumin levels were significantly lower in the BSC (p<0.001).

Living situation

In the BSC group, 16 patients (66.7%) lived with their families, while eight patients (33.3%) lived alone or in a care facility. In the non-BSC groups, 94 patients (84.7%) lived with their families, and 17 patients (15.3%) lived alone. The difference in living situation between the two groups was not statistically significant (χ2=4.25, df=1, p=0.99, φ=0.18; Table 5).

**Table 5:** Living situation BSC: best supportive care

Living situation	BSC group	Non-BSC group	p-value
	n=24 (%)	n=111 (%)	
Lived with family	16 (66.7)	94 (84.7)	0.99
In a care facility	5 (20.8)	0 (0)	
Lived alone	3 (12.5)	17 (15.3)	

Rationale for BSC selection

Poor general condition was the primary reason for selecting BSC in 14 patients (58.3%). Although 10 patients (41.7%) were considered eligible for definitive treatment - surgery in seven patients (29.2%) and chemoradiotherapy in three patients (12.5%) - BSC was ultimately chosen based on the patient preference.

Survival rates

**Figure 2:**
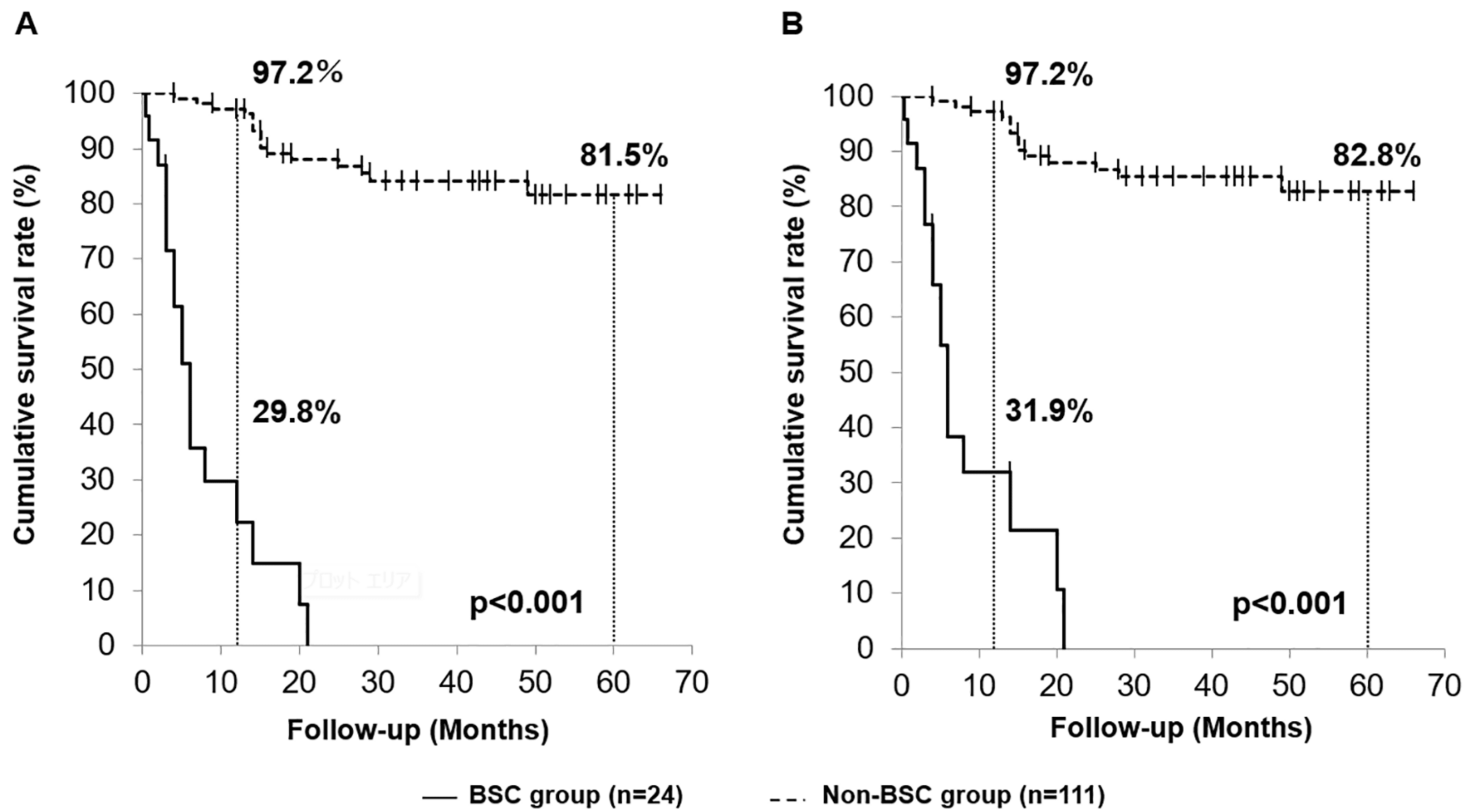
Survival rate (A) The cumulative one-year OS rate in the BSC group is 29.8%, while the cumulative one-year and five-year OS rates in the non-BSC group are 97.2% and 81.5%, respectively. A statistically significant difference is observed between the two groups (p<0.001). (B) The cumulative one-year DSS rate in the BSC group is 31.9%, whereas the cumulative one-year and five-year DSS rates in the non-BSC group are 97.2% and 82.8%, respectively. A statistically significant difference is observed between the two groups (p<0.001). OS: overall survival; DSS: disease-specific survival; BSC: best supportive care

The cumulative one-year OS rate in the BSC group was 29.8%, whereas the cumulative one-year and five-year OS rates in the non-BSC group were 97.2% and 81.5%, respectively. This difference was statistically significant (p<0.001; Figure 2A). The cumulative one-year DSS rate in the BSC group was 31.9%, while the cumulative one-year and five-year DSS rates in the non-BSC group were 97.2% and 82.8%, respectively. A statistically significant difference was also observed between the two groups (p<0.001; Figure 2B).

Multiple logistic regression analysis

The results of the logistic regression analysis are exhibited in Table 6. Univariate regression identified age, stage classification (I/II vs. III/IV), ECOG-PS (0-1 vs. 2-4), psychiatric disorder, and albumin level as factors significantly associated with BSC (p<0.0001). Multivariate logistic regression revealed that age (odds ratio (OR): 1.20, 95% CI: 1.10-1.36; p=0.0008), stage classification (I/II vs. III/IV) (OR: 13.79, 95% CI: 2.34-137.75; p=0.0026), psychiatric disorder (OR: 8.73, 95% CI: 1.38-75.43; p=0.021), albumin level (OR: 0.03, 95% CI: 0.002-0.24; p=0.003) were significant factors in BSC.

**Table 6:** Results of univariate and multivariate analyses in risk factors for BSC BSC: best supportive care; ECOG-PS: Eastern Cooperative Oncology Group Performance Status

Variables	Univariate	Multivariate	
	p-value	p-value	Odds rate (95% CI)
Age	<0.0001	0.0008	1.20 (1.10-1.36)
Sex	0.19	―	―
Stage Classification (Ⅰ-Ⅱ vs. Ⅲ-Ⅳ)	<0.0001	0.0026	13.79(2.34-137.75)
ECOG-PS (0-1 vs. 2-4)	<0.0001	0.24	3.27 (0.47-31.66)
Cardiovascular disease	0.35	―	―
Psychiatric disorder	<0.0001	0.021	8.73 (1.38-75.43)
Endocrine metabolic disease	0.98	―	―
Digestive system disease	0.36	―	―
Neutrophil-to-lymphocyte ratio	0.52	―	―
Albumin level	<0.0001	0.003	0.03 (0.002-0.24)
Living situation (lived with family vs. in a care facility, lived alone）	0.052	―	―

Patient aged ≥ 75 years

The comparison between the BSC and non-BSC group for patients aged ≥75 years is depicted in Table 7. In the BSC group, 23 patients (95.8%) were aged ≥75 years, compared with 34 (30.6%) in the non-BSC group. The proportion of patients aged ≥75 years was higher in the BSC group. There were more women in both groups. Analysis of stage classification (χ2=10.97, df=1, p<0.001, φ=0.43), ECOG-PS (χ2=13.71, df=1, p<0.001, φ=0.49), and albumin levels (p<0.001) demonstrated statistically significant differences between the two groups in this age category. The results of the logistic regression analysis for ≥75 years are summarized in Table 8. Univariate regression identified stage classification (I/II vs. III/IV), ECOG-PS (0-1 vs. 2-4), psychiatric disorder, and albumin level as significant factors associated with BSC (p<0.001). Multivariate logistic regression revealed that stage classification (I/II vs. III/IV) (OR: 19.08, 95% CI: 2.51-321.07, p=0.0027), and albumin level (OR: 0.003, 95% CI: 9.0×10^-6^-0.098, p=0.01) were factors in BSC.

**Table 7:** Patient aged ≥ 75 years data BSC: best supportive care; ECOG-PS: Eastern Cooperative Oncology Group Performance Status

Variables	BSC group	Non-BSC group	p-value
	n=23 (95.8%)	n=34 (30.6%)	
Sex			
Male	10 (43.5)	15 (44.1)	
Female	13 (56.5)	19 (55.9)	
Male:Female	1:1.3	1:1.27	
Underlying diseases			
Cardiovascular disease	12 (52.2)	23 (67.6)	
Psychiatric disorder	10 (43.5)	3 (8.8)	
Endocrine metabolic disease	10 (43.5)	15 (44.1)	
Cerebrovascular disease	7 (30.4)	2 (5.9)	
Digestive system disease	3 (13)	11 (32.4)	
Respiratory disease	3 (13)	5 (6.3)	
Autoimmune disease	0 (0)	3 (14.7)	
Others	20 (87)	26 (76.5)	
Stage			
Ⅰ	0 (0)	10 (29.4)	<0.001
Ⅱ	4 (17.4)	11 (32.4)	
Ⅲ	2 (8.7)	4 (11.8)	
ⅣA	12 (52.2)	8 (23.5)	
ⅣB	4 (17.4)	1 (2.9)	
ⅣC	1 (4.3)	0 (0)	
ECOG-PS			
0	2 (8.7)	21 (61.8)	<0.001
1	10 (43.5)	11 (32.3)	
2	2 (8.7)	2 (5.9)	
3	6 (26.1)	0 (0)	
4	3 (13)	0 (0)	
NLR	3.03 (±1.05)	2.52 (±1.43)	0.92
Albumin level	3.54 (±0.29)	4.04 (±0.32)	<0.001
Living situation			
Lived with family	16 (69.6)	28 (82.4)	0.26
In a care facility	5 (21.7)	0 (0)	
Lived alone	2 (8.7)	6 (17.6)	

**Table 8:** Results of univariate and multivariate analysis in risk factors for BSC aged ≥ 75 years ECOG-PS: Eastern Cooperative Oncology Group Performance Status

Variables	Univariate	Multivariate	
	p-value	p-value	Odds rate (95% CI)
Sex	0.96	―	―
Stage classification (Ⅰ-Ⅱ vs. Ⅲ-Ⅳ)	0.0006	0.0027	19.08 (2.51-321.07)
ECOG-PS (0-1 vs. 2-4)	0.0002	0.2	3.57 (0.51-38.17)
Cardiovascular disease	0.24	―	―
Psychiatric disorder	0.0021	0.17	3.91 (0.55-37.80)
Endocrine metabolic disease	0.96	―	―
Digestive system disease	0.087	―	―
Neutrophil-to-lymphocyte ratio	0.16	―	―
Albumin level	<0.0001	0.01	0.003 (9.0×10^-6^-0.098)
Living situation (lived with family vs. in a care facility, lived alone)	0.26	―	―

## Discussion

This study investigated the clinical characteristics of patients with OSCC whose initial treatment strategy was BSC and the factors influencing their treatment policy decisions. Among 135 patients with primary OSCC, 24 (17.8%) received BSC, and 111 (82.2%) underwent other treatments (non-BSC). Cheraghlou et al. [4] investigated 36,261 patients with oral cancer and reported that 356 (0.98%) did not choose any treatment. Patients ≥ 75 years and those with advanced T3 or T4 often declined definitive treatment [4]. In the BSC group, 95.8% (n=23) were aged ≥75 years, compared to 30.6% (n=34) in the non-BSC group, indicating BSC patients tended to be older.

The male-to-female ratio was 1:1.2 in the BSC group and 1:0.7 in the non-BSC group, suggesting a higher proportion of female patients in the BSC group. While OSCC is generally more prevalent in males, the predominance of female patients in the BSC group in this study may reflect a higher mean age in this group (83.9 years) compared to the non-BSC group (67.4 years). In Japan, the average life expectancy is 81.4 years for men and 87.5 years for women [5]. Additionally, among individuals aged ≥80 years, women comprise 6.1% of the population, compared to 3.5% for men [6]. This demographic distribution likely explains the predominance of female patients in the older BSC group.

The most common primary site in the BSC group was the lower gingiva (n=7, 29.1%), followed by the tongue (n=6, 25%). Conversely, in the non-BSC group, the tongue was predominant (n=58, 52.3%), followed by the lower gingiva (n=17, 15.3%). Although the tongue is typically the most frequent site for OSCC [7], in the BSC cohort, gingival cancers (lower + upper) represented 41.6% (n = 10), making the gingiva the most common site. This may be related to the known higher incidence of gingival cancer is higher in older people [8] and is consistent with the older age distribution of the BSC group.

Compared to the non-BSC group, the BSC group had a higher proportion of advanced stage III/IV cases. Furthermore, 83.3% (n=20) of patients in the BSC group had advanced disease, suggesting that a desire to avoid highly invasive treatments possibly contributed to the selection of BSC.

Regarding general condition and living situation, 13 patients (54.2%) in the BSC group and 108 patients (97.3%) in the non-BSC group had an ECOG-PS score <2, indicating no activity restrictions in daily life. Among the 13 patients in the BSC group, four (30.8%) were in a care facility or living alone, and all were stage IV. In the non-BSC group, 17 patients (15.7%) lived alone, of whom eight (7.4%) were stage IV. Eleven patients (45.8%) in the BSC group and three patients (2.7%) in the non-BSC group had an ECOG-PS score ≥2. Among the 11 patients in the BSC group, four (36.4%) were in a care facility, and one patient (4.2%) was stage III, whereas three patients (12.5%) were stage IV. These results suggest that, in addition to the progression of ECOG-PS, social isolation, such as living alone or in a facility, may delay seeking medical care, leading to the progression of the disease and the selection of BSC.

In both groups, cardiovascular disease was the most common observed underlying disease. Psychiatric disorder was more prevalent in the BSC group, while endocrine and metabolic disease was notable in the non-BSC group. In older patients and those with psychiatric disorders, understanding patient values and preferences can be challenging [9]. Therefore, underlying diseases may have influenced policy decisions in the BSC group.

The NLR is recognized as a prognostic marker in various cancers and a potential prognostic indicator in head and neck squamous cell carcinoma [10-12]. Chen et al. [13] reported that patients with OSCC exhibiting a pretreatment NLR of >3.66 experienced significantly decreased overall survival compared to those with an NLR of <1.94, indicating that a higher NLR is associated with poor prognosis. The mean pretreatment NLR for patients in OSCC reportedly ranges from 2.48 to 3.68 [14-16]. In the current study, the mean NLR at initial examination was 3.09±1.06 and 2.76±2.26 in the BSC and non-BSC groups, respectively. While no statistically significant difference was found between the two groups, the relatively higher NLR in the BSC group may reflect the higher proportion of advanced cases and suggest a poorer prognosis.

Serum albumin levels are also considered prognostic factors in head and neck cancers. Medow et al. [17] reported poor prognosis in stage IV or recurrent cases with serum albumin levels <3.85 g/dL. In the present study, the mean serum albumin level at the initial examination was 3.51±0.32 g/dL in the BSC group and 4.17±0.40 g/dL in the non-BSC group, with the difference being statistically significant. The mean serum albumin level in the BSC group was <3.85 g/dL, suggesting a poor prognosis based on the patients’ nutritional status.

Poor general condition was the primary reason for selecting BSC in 14 patients (58.3%). The remaining ten patients (41.7%) were medically eligible for definitive treatment, but ultimately opted for BSC on personal preference. Of these 10 patients, five were males and five were females; four of the males were aged ≥81 years, and all of the females were aged ≥86 years. The decision may reflect a psychological tendency toward “acceptance of death,” which has been observed in older individuals approaching average life expectancy.

In this study, the one-year OS and DSS rates in the BSC group were 29.8% and 31.9%, respectively. Contrarily, the non-BSC group demonstrated markedly better outcomes, with one-year and five-year OS rates of 97.2% and 81.5% and one-year and five-year DSS rates of 97.2% and 82.8%, respectively. Kowalski et al. [18] reported a one-year OS rate of 15.8% among patients with untreated oral cancer, 93.2% of whom were diagnosed at stage IV, and 86.1% had an ECOG-PS score of 2 or higher. Similarly, in our study, 75% (n=18) of the BSC patients were stage IV, and 45.8% (n=11) had an ECOG-PS score ≥2. These results indicate that the progression of the disease stage and general condition significantly influence the prognosis.

Multivariate analysis revealed that age, stage classification, psychiatric disorder, and albumin level were more significant factors of BSC. As the BSC group tended to be older than the non-BSC group, a subgroup analysis was conducted in patients aged ≥75 years. Even within this older population, stage classification and albumin level remained factors of BSC. The progression of the stage was shown to be an important factor in BSC. It is possible that, if cancer had been detected earlier or if less invasive treatment options were available, some patients would have chosen aggressive treatment.

Treatment strategy decisions for cancer patients depend on factors such as the disease stage; physical, psychological, and social background; and preferences of patients and their families [19]. In this study, 14 patients (58.3%) in the BSC group were of advanced age, with average age of 83.7 years, and exhibited comorbidities such as psychiatric disorder (n=9, 64.3%), cardiovascular disease (n=5, 35.7%), cerebrovascular disease (n=5, 35.7%), and high levels of care dependency. These multiple factors raised concerns that active treatment could further deteriorate their general condition. Given the high risk of cancer treatment, and the availability of multiple treatment options, shared decision-making (SDM) between patients and healthcare professionals is important [19]. Accurately understanding the values and preferences of older patients can be particularly challenging [20]. These individual and systemic factors may have influenced treatment decisions and may have been a barrier to early consultation at a medical institution and active treatment selection. SDM may not have been adequately implemented in BSC patients, highlighting the need to promote SDM to determine treatment policies for cancer patients.

Older patients often avoid invasive treatments and may face environmental or systemic barriers to early access to medical care. Early detection and timely treatment are necessary to reduce the number of patients opting for BSC in the future. Local dental check-ups and screenings during home-visit medical care can support early detection. Educating healthcare providers and introducing simple non-invasive diagnostic tools, such as oral cancer screenings using salivary biomarkers [21,22] and fluorescence visualization devices [23,24], as previously reported, can enhance early detection. Conversely, as comprehensive care to improve the QOL of BSC patients, improving end-of-life care remains a key concern. Depending on the patient's general condition and patient background, a collaborative dialogue involving the patient and their family to create a care plan that reflects patient values and preferences is essential. In the future, early detection and treatment should be considered not only by medical professionals but also by caregivers, occupational therapists, and community members. Additionally, interdisciplinary collaboration with physical therapists, nutritionists, care managers, and social workers is essential to provide personalized support, helping each patient receiving BSC, the end of life in a manner that is meaningful and dignified.

Limitations

This study has certain limitations. First, the sample size was small - 24 and 111 patients in the BSC and non-BSC groups, respectively - limiting the generalizability of the findings. Additionally, detailed information regarding patients’ background and reasons for delays in seeking medical care could not be thoroughly analyzed. Future prospective studies with larger, more diverse cohorts are needed to validate these findings.

## Conclusions

This study identified advanced age, stage classification, presence of psychiatric disorders, and low serum albumin levels as significant risk factors associated with the selection of BSC as the initial treatment strategy. Particularly noteworthy is the tendency among older patients to decline curative treatment when it is associated with high invasiveness. Conversely, early identification of the lesion may allow for the consideration of less invasive therapeutic options, thereby potentially increasing treatment acceptance.

These findings underscore the importance of early diagnosis and the need to incorporate patients’ social and functional contexts into treatment planning. In advanced OSCC, where treatment options are often limited, promoting SDM and developing personalized care plans that reflect medical feasibility and patient values are essential.
